# Seed coat color genetics and genotype × environment effects in yellow beans via machine‐learning and genome‐wide association

**DOI:** 10.1002/tpg2.20173

**Published:** 2021-11-24

**Authors:** Rie Sadohara, Yunfei Long, Paulo Izquierdo, Carlos A. Urrea, Daniel Morris, Karen Cichy

**Affiliations:** ^1^ Dep. of Plant, Soil and Microbial Sciences Michigan State Univ. 1066 Bogue St. East Lansing MI 48824 USA; ^2^ Dep. of Electrical and Computer Engineering Michigan State Univ. 428 S Shaw Ln. East Lansing MI 48824 USA; ^3^ Panhandle Research & Extension Center Univ. of Nebraska 4502 Ave. I Scottsbluff NE 69361 USA; ^4^ USDA–ARS, Sugarbeet and Bean Research Unit 1066 Bogue St. East Lansing MI 48824 USA

## Abstract

Common bean (*Phaseolus vulgaris* L.) is consumed worldwide, with strong regional preferences for seed appearance characteristics. Colors of the seed coat, hilum ring, and corona are all important, along with susceptibility to postharvest darkening, which decreases seed value. This study aimed to characterize a collection of 295 yellow bean genotypes for seed appearance and postharvest darkening, evaluate genotype × environment (G × E) effects and map those traits via genome‐wide association analysis. Yellow bean germplasm were grown for 2 yr in Michigan and Nebraska and seed were evaluated for L*a*b* color values, postharvest darkening, and hilum ring and corona colors. A model to exclude the hilum ring and corona of the seeds, black background, and light reflection was developed by using machine learning, allowing for targeted and efficient L*a*b* value extraction from the seed coat. The G × E effects were significant for the color values, and Michigan‐grown seeds were darker than Nebraska‐grown seeds. Single‐nucleotide polymorphisms (SNPs) were associated with L* and hilum ring color on Pv10 near the *J* gene involved in mature seed coat color and hilum ring color. A SNP on Pv07 associated with L*, a*, postharvest darkening, and hilum ring and corona colors was near the *P* gene, the ground factor gene for seed coat color expression. The machine‐learning‐aided model used to extract color values from the seed coat, the wide variability in seed morphology traits, and the associated SNPs provide tools for future breeding and research efforts to meet consumers’ expectations for bean seed appearance.

## Core Ideas

- Postharvest darkening in yellow beans is associated with *P* gene.
- Environment influences yellow seed coat color independent of postharvest darkening.
- Imaging combined with machine learning is a useful way to measure bean seed coat color.
- Genotype and environmental differences in bean seed coat color are detectable via imaging.

**Abbreviations**
BLUE: best linear unbiased estimate
G × E: genotype × environment
PC: principal component
QTL: quantitative trait loci
SNP: single‐nucleotide polymorphism
YBC: Yellow Bean Collection

## 1 INTRODUCTION

Common bean (*Phaseolus vulgaris* L.) is a nutrient‐dense food consumed worldwide, with an annual world production of 29 Tg (average of 2016–2019; FAOSTAT, 2021). Beans serve as an excellent source of protein, dietary fiber, and micronutrients and offer numerous health benefits including prevention and control of cardiometabolic diseases and certain types of cancer (Hayat et al., 2014; Messina, 2014). The seed appearance of beans is diverse with various shapes, sizes, colors, and patterns, the combinations of which form specific bean market classes (seed types) (González et al., 2006; Uebersax & Siddiq, 2012; Wortmann et al., 1998). Therefore, bean breeders develop cultivars with morphological characteristics that meet the market demands in their target regions (Beebe, 2020; Castellanos et al., 1996; O. Voysest et al., 1994). In addition to the primary seed color and pattern, the hilum ring and corona color are also essential parts of seed appearance.

Since early in the 20th century, extensive research has been conducted to identify genes responsible for producing bean seed coat color. Prakken (1972, 1974) consolidated initial findings and developed a model for color expression in the bean seed coat, which, to date, is generally accepted. The model groups color‐related genes into three categories: a ground factor gene, color genes, and color‐modifying genes. A ground factor gene, *P*, is necessary for any seed color expression, and genotypes with homozygous recessive *p* produce a white seed coat. The *P* gene was later found to encode a transcription factor required for flavonoid biosynthesis (McClean et al., 2018). Color genes include *C*, *Z*, and *J* genes. Multiallelic *C* locus is tightly linked with other genes, forming ‘complex C locus’, which are involved in different color and pattern expressions (Bassett, 2007). The *Z* gene gives a brown hilum ring and is also involved in partly colored seed coat (Bassett et al., 1999). The *J* gene is involved in multiple traits such as seed coat color formation during maturation, a brown hilum ring, seed coat shininess, and postharvest darkening (Bassett, 1996; Prakken, 1970). Postharvest darkening refers to a phenomenon where seed color of some market classes darkens over time during storage. Postharvest darkening is an important trait for bean producers because consumers consider darkened seeds as old and long cooking, so darkened seeds may be of lower economic value (Junk‐Knievel et al., 2008). Previous studies have shown that two unlinked interacting genes control postharvest darkening, *J* and *sd*, with the *J* genotype presenting postharvest darkening, *jj* genotype showing no darkening, and recessive *sdsd* paired with *J* showing slow darkening (Elsadr et al., 2011; Junk‐Knievel et al., 2008). Gene *sd* has been confirmed to be an allele of the ground factor *P* and is denoted as *p^sd^* (Islam et al., 2020). Color‐modifying genes do not confer a color by themselves but intensify color expression of the color genes. Color‐modifying genes include *G* for yellow‐brown, *B* for greenish yellow‐brown, *V* for purple to black, *Rk* for recessive red, *R* for dark red, and *Gy* for greenish‐yellow (Bassett, 2002; Bassett et al., 2010; Beninger & Hosfield, 1999). The mechanism of color expression is complicated; some of the genes are multiallelic, and they interact with each other to collectively determine the colors and patterns of seed coat, hilum ring, corona, pods, and flowers (Bassett, 2007).

Although seed coat colors could be treated as a qualitative trait controlled by major genes, more recent research has quantitatively measured colors using CIE L*a*b* values (CIE International Commission on Illumination, 2004). The CIE L*a*b* expresses colors with three values, L*, a*, and b*, which measure lightness, greenness–redness, and blueness–yellowness, respectively defined as axes in a three‐dimensional color space. L*a*b* values are perceptually uniform, meaning that the degree of difference between two colors corresponds to the Euclidean distance of them in the L*a*b* space (León et al., 2006). Expressing colors using numerical values enables an objective measurement of color rather than a subjective description. Traditionally, colorimeters were used to measure color values, but image‐based, computer‐vision technology has also been used (Wu & Sun, 2013). Image‐based color measurement provides a better representation of samples because it can obtain L*, a*, and b* values from each pixel of an image, and a larger surface area can be sampled compared with conventional colorimeters (León et al., 2006). Depending on the type of samples, images can contain pixels that do not represent the specimen. In the case of bean seed coat color measurement, the nontarget pixels would be the white hilum, a hilum ring, and corona of seeds as well as the imaging background and light reflection. Measuring color values without excluding those could result in differences in color values because of a darker hilum ring and corona despite the same seed coat color of two bean samples. Machine learning presents a potential to segment seed coat from nontargets in bean seed images because machine learning has been used to separate target features from nontargets of plant images (Amatya et al., 2016; Ferentinos, 2018; Grinblat et al., 2016).

**Core Ideas**

- Postharvest darkening in yellow beans is associated with *P* gene.
- Environment influences yellow seed coat color independent of postharvest darkening.
- Imaging combined with machine learning is a useful way to measure bean seed coat color.
- Genotype and environmental differences in bean seed coat color are detectable via imaging.

Numerous polyphenolic compounds exist in the seed coat of common bean such as flavonoids (Yang et al., 2018), which are considered to impart bean seed color (Beninger et al., 1998; Feenstra, 1960). Flavonoids present in the bean seed coat can be subclassified into flavonols, flavanols, and anthocyanidins (Reddy et al., 1985). Anthocyanidins are glycosides of anthocyanins, including cyanidin, pelargonidin, and delphinidin, and are found in pink, red, and black bean seeds (Rodríguez Madrera et al., 2020; Takeoka et al., 1997). Proanthocyanidins (condensed tannins) are dimers or polymers of flavanols, including catechin and epicatechin (Dixon et al., 2005). Proanthocyanidins produce brown deposits upon oxidation in pinto bean seeds and are associated with postharvest darkening (Beninger & Hosfield, 2003; Marles et al., 2008). Proanthocyanidins are also involved in plant defense against pests and pathogens (Islam et al., 2003; Winkel‐Shirley, 2001). In addition to seed coat color and plant defense responses, the concentrations of polyphenolic compounds influence quality attributes of beans such as cooking time, digestibility, and iron bioavailability (Bressani & Elías, 1979; Elia et al., 1997; Petry et al., 2010; Tako et al., 2014).

**FIGURE 1:**
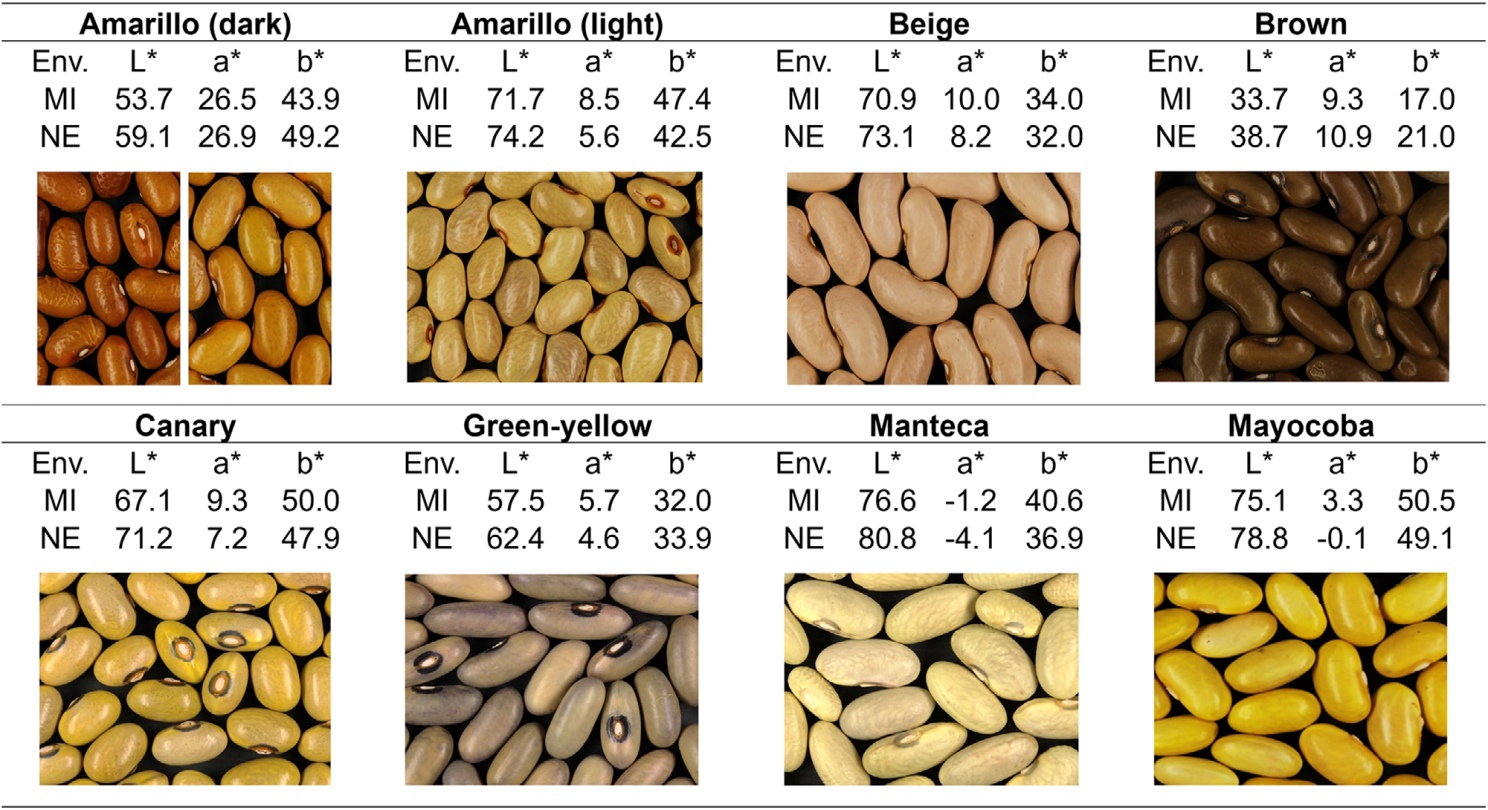
Eight major seed types in the Yellow Bean Collection (YBC) and their 2‐yr average L*a*b* values in each environment (Env.). MI, Michigan; NE, Nebraska

Yellow bean is an important market class in eastern and southern Africa and Latin America (Voysest, 2012; Wortmann et al., 1998). Yellow bean is diverse in seed morphology (Figure 1), and at least a dozen yellow‐seeded market classes exist in Latin America alone (Voysest, 2012). One of the valuable attributes of some yellow bean cultivars is that they do not exhibit postharvest darkening. The susceptibility to postharvest darkening of yellow bean is mainly unknown except that genetic variability and a quantitative trait loci (QTL) for postharvest darkening near the *J* gene were reported among Manteca and green–yellow types and their progeny population (Bassett et al., 2021). Postharvest darkening is known to occur in carioca bean, which have light brown stripes on a cream background similar to pinto bean (Junk‐Knievel et al., 2008); therefore, some yellow bean cultivars may be susceptible to postharvest darkening.

Evidence is accumulating that a lemon‐yellow seeded type, Manteca, is a promising breeding material because of its high digestibility, short cooking time, and high iron bioavailability (Cichy et al., 2015; Engleright et al., 1999; Hart et al., 2020; Hooper et al., 2016; J. Wiesinger et al., 2018; Wiesinger et al., 2019). Other yellow bean cultivars’ convenience and nutritional qualities have yet to be assessed despite their wide morphological diversity. Therefore, the Yellow Bean Collection (YBC) has been assembled with yellow‐seeded *P. vulgaris* germplasm collected globally (Supplemental Figure S1). Although high heritability estimates (73–99%) were reported for color values with populations derived from yellow‐seeded beans (Arns et al., 2018; Bassett et al., 2021; Possobom et al., 2015; Ribeiro et al., 2019), growing conditions could influence their seed coat colors (Kelly et al., 2021; Possobom et al., 2015). Thus, the genotype × environment (G × E) effects on the seed color of the YBC should be evaluated to develop bean cultivars that meet the consumers’ expectations in various bean production regions. Quantitative characterization of the seed coat colors of the YBC would enable an objective evaluation of their appearance, an important characteristic to consumers.

The objectives of this study were to (a) assess the genetic diversity of the YBC of 295 yellow bean genotypes for seed coat color values via a machine‐learning aided procedure; (b) to assess the postharvest darkening of the YBC; and (c) investigate the genetic control and the G × E on these traits to aid breeding of beans with desired morphological, nutritional, and culinary characteristics.

## 2 MATERIALS AND METHODS

### 2.1 Plant materials

A yellow bean diversity panel, the YBC, comprising 295 genotypes, was used in this study. The YBC was grown in randomized complete block design with two field replications in two environments, Michigan and Nebraska, USA, in 2018 and 2019. The number of genotypes phenotyped for color, postharvest darkening, and hilum ring and corona colors are shown in Supplemental Table S1.

### 2.2 CIE L*a*b* of bean seed coat

**FIGURE 2:**
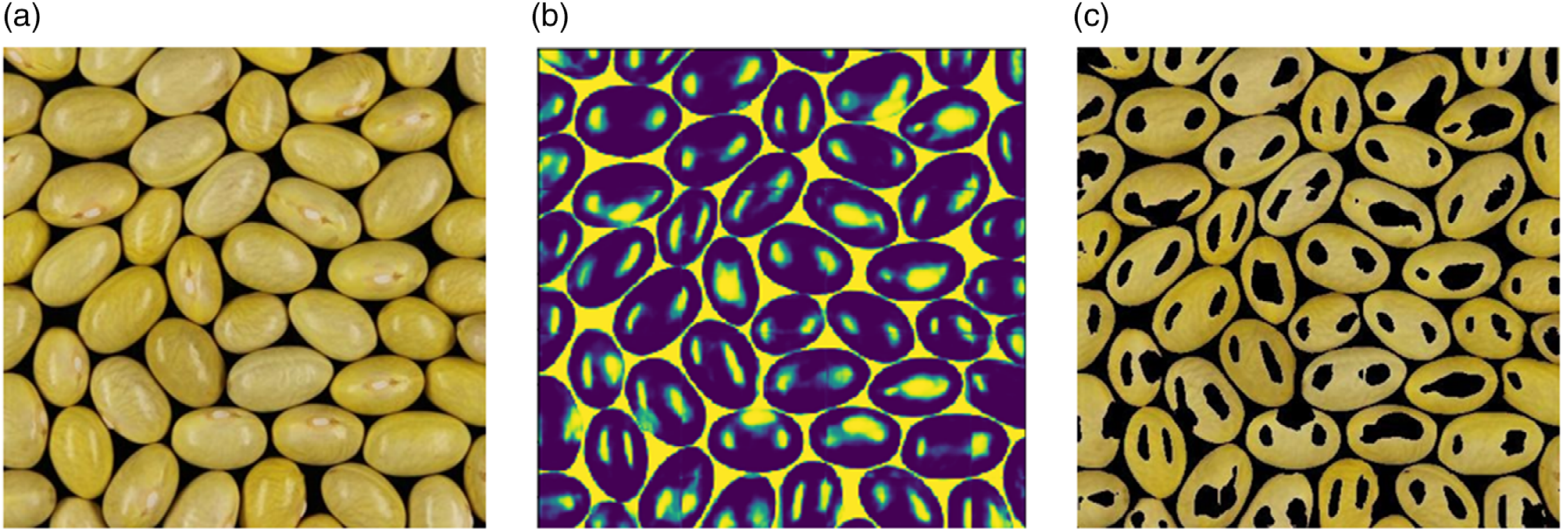
(a) Original, (b) segmented by convolutional neural networks, and (c) masked images. The probability of each pixel of being seed coat is color‐coded from dark blue (high) to yellow (low) in (b)

The YBC contained solid‐colored and patterned seed types, but the solid‐colored types were used for seed coat color measurements. The genotypes used for color measurements were as follows: 37 Amarillo dark, 44 Amarillo light, six beige, seven brown, 83 Canary, six green–yellow including Njano, 11 Manteca, 56 Mayocoba, and three white genotypes. One layer of solid‐colored, intact whole seeds were placed in a 70‐ by 70‐mm box with matte black walls. Images of bean seeds from each of the two field replications were taken by using an image acquisition system described by Mendoza et al. (2017) with the shutter speed set to 1/100 s. To extract bean color from captured images via machine learning, a binary pixel classification was defined in which pixels representative of bean color are one category, and the remaining pixels, that is, background, hilum ring, corona, and specular reflection areas, are the second category. The pyramid convolutional neural network described in Long et al. (2019) to detect bean seed coat splits was retrained for this color pixel segmentation task. To train the network, semantic labels for 24 images (including 17 images for training and seven for validation) of various bean types and colors within the YBC were created manually and augmented by rotation (at 30° intervals) and flipping. The training was performed for eight epochs by using ADADELTA (Zeiler, 2012) as the optimizer with a learning rate of one and pixel‐wise cross‐entropy as the loss function. The network achieved 0.95 average precision in bean pixel classification on a validation set and so was able to effectively eliminate nondesired pixels (identified as pixels with predicted probability for the second category larger than 0.3) from images as illustrated in Figure 2. The source code, annotated images, and trained model are available online (https://github.com/longyunf/extract‐bean‐color).

Bean images were downsampled to 432 by 432 pixels using a custom MATLAB script (MATLAB, 2020), and the segmentation model was applied. The CIE L*, a*, and b* values were extracted from valid pixels in the images by scikit‐image in Python (van der Walt et al., 2014). The L*a*b* values were calibrated using an image of Macbeth color checker chart (X‐Rite) taken under the same conditions as the YBC at the start of image acquisition each year. The 24 color patches in the Macbeth color checker chart image were cut out into 100‐ by 100‐pixel squares using ImageJ 1.52v (Schneider et al., 2012), and the L*a*b* values were extracted from each patch by the scikit‐image in Python. The extracted L*a*b* values and the standard values of the patches were used to build a partial least squares model by the PLS package (Mevik et al., 2020) in R (R Core Team, 2017). Specifically, SIMPLS, a faster and improved partial least squares model (de Jong, 1993) with three components, was used to calibrate L*a*b* values of the YBC bean images, and calibration was carried out each year to accommodate potential changes in lighting intensity over the 1‐yr period. The L*a*b* color values were evaluated for 252 genotypes in Michigan in 2018, 250 in Michigan 2019, 209 in Nebraska 2018, and 195 in Nebraska, depending on the seed availability and adaptation (Supplemental Table S1).

To evaluate the difference in seed coat colors between the environments, ΔL*, Δa*, and Δb* were calculated as the mean L*a*b* values in Michigan subtracted by those in Nebraska for each genotype. The ΔE was computed as follows: $\Delta E=\sqrt[{2}]{\Delta L^{*2}+\Delta a^{*2}+\Delta b^{*2}}$.

### 2.3 Postharvest darkening

Postharvest darkening of MI2019 seeds (both solid‐colored and patterned) was evaluated via an ultraviolet test developed by Junk‐Knievel et al. (2007). In summary, ∼10 seeds per sample per field replication were placed in a clear plastic seed tray. The tray held 60 samples in total. Trays containing seeds were placed ∼10 cm under a UV light for 96 h. After that time period, the seeds were scored for darkening as 0 for non‐ or slow darkening and 1 for darkening. Some of the dark‐seeded genotypes, such as brown and Amarillo (dark) types, were excluded from this evaluation.

### 2.4 Hilum ring and corona colors

The hilum ring and corona colors were evaluated of the MI2019 seeds (both solid‐colored and patterned). The hilum ring colors were classified into two categories: 0 for yellow and 1 for dark. The corona colors were classified into two categories: 0 for light or yellow and 1 for dark.

### 2.5 Statistical analyses

The L*a*b* values were transformed using the Box–Cox method (Box & Cox, 1964) by using the MASS package (Venables & Ripley, 2002) in R. The genotype, environment, and year effects on the color values were estimated by the following mixed linear model: $Y_{ijkl}=\mu +G_{i}+E_{j}+Y_{k}+EY_{jk}+GE_{ij}+GY_{ik}+\mathrm{GE}Y_{ijk}+B{\left(\mathrm{EY}\right)}_{jkl}+\varepsilon_{ijkl}$, where *Y_ijkl_* is the phenotypic value of the *i*th YBC genotype grown in the *l*th block of the *j*th environment in the *k*th year; μ is the grand mean; *G_i_* is a random effect of the *i*th genotype; *E_j_* and *Y_k_* are fixed effects of the *j*th location and the *k*th year, respectively; EY*_jk_*, GE*_ij_*, and GY*_ik_* are two‐way interaction terms; GEY*_ijk_* is a three‐way interaction term; *B*(EY)*_jkl_* is a random effect of the *l*th block nested in the *j*th environment and the *k*th year; and ε*_ijkl_* is the error term. The variance components of the model were used to estimate the broad‐sense heritability of the phenotypic values (Fehr, 1987). To minimize the environment and year effect, best linear unbiased estimate (BLUE) was calculated for the L*, a*, and b* values by setting the genotype as a fixed effect and all other terms as random effects using the emmeans package (Lenth, 2021) in R. All the models were fitted using the lme4 (Bates et al., 2015) and the lmerTest (Kuznetsova et al., 2017) packages in R. The BLUE values were used for principal component (PC) analysis and genome‐wide association analysis. Principal component analysis was performed using prcomp function and was visualized using the factoextra package (Kassambara & Mundt, 2020) in R.

### 2.6 Genome‐wide association analysis

Genome‐wide association analyses were carried out using the BLUEs of the CIE L*a*b*, coded darkening phenotype (0, nondarkening; 1, darkening), hilum ring color (0, yellow; 1, dark), and corona color (0, light or yellow; 1, dark). In total, 253 genotypes for L*a*b*, 206 genotypes for postharvest darkening, and 258 genotypes for hilum ring and corona colors were used. The 295 genotypes of the YBC were sequenced via genotyping‐by‐sequencing technology (Elshire et al., 2011) with 150‐bp single‐end reads. The reads were aligned to common bean genome ver. 2.0 (Phytozome, https://phytozome.jgi.doe.gov/) using NGSEP (Duitama et al., 2014), generating a base SNP set of 417,142 SNPs. The base SNP set was filtered to include the 253, 206, and 258 phenotyped individuals. The SNPs were selected if they were biallelic, had no heterozygous calls, had a genotyping quality score >40, were not on scaffolds, were outside the repetitive (nonunique alignment) regions of the common bean genome (G19833, v2; Lobaton et al., 2018), had <20% missing data, and had >5% minor allele frequency. As a result, 2,277 SNPs for L*a*b*, 2,278 SNPs for postharvest darkening, and 2,315 SNPs for hilum ring and corona colors were retained and used for genome‐wide association analyses with the GAPIT package (Lipka et al., 2012) in R. Bayesian‐information and linkage‐disequilibrium iteratively nested keyway method was used to detect associations between the phenotypes and the genotypes (Huang et al., 2019). A false discovery rate at α = 0.05 was used to call associations significant. Quantile–quantile plots and Manhattan plots were generated by using the CMplot package (Yin, 2020) in R.

## 3 RESULTS AND DISCUSSION

### 3.1 Phenotypic diversity of seed coat, hilum ring, and corona colors and postharvest darkening

Employing the machine‐learning technology, seed coat was successfully separated from hilum and corona of seeds, black background, and light reflection after feeding 24 hand‐labeled images for training. The model correctly distinguished pixels of seed coat of >1,800 images of the YBC genotypes without manual operation. The automation allows for more accurate measurement of seed coat color and saves a tremendous amount of time and labor that would have been spent on the manual processing of images. Automated separation of seed coat and hilum ring and corona areas are substantial because they are all under different genetic controls (Bassett, 2007). Bean seed colors have been measured traditionally with colorimeters (Arns et al., 2018; Erfatpour et al., 2018; Possobom et al., 2015) and more recently with ImageJ (Bassett et al., 2021; Bornowski et al., 2020; Varga et al., 2019), but the seed coat, hilum ring, and corona colors were not separated. Thus, this study has provided a novel, automated method to obtain color values from seed coat pixels.

The L* values range from 0 to 100 with 100 being the lightest; positive a* values indicate redness while negative a* indicates greenness; and positive b* indicates yellowness, while negative b* indicates blueness (León et al., 2006). A wide diversity in the seed coat color values were observed among the YBC genotypes: L* values ranged from 27.6 to 86.0, a* values from −5.9 to 35.0, and b* values from 11.9 to 63.6 (Table 1). The 2‐yr average of L*a*b* values were highly correlated between the environments, but the correlation coefficient was slightly lower for the b* values (*R* = 0.88, *p* < .001), which measures yellowness, than L* and a* (*R* = 0.98, *p* < .001 for both; Supplemental Figure S2). The L* and a* values were negatively correlated (*R* = −0.69 in Michigan and *R* = −0.73 in Nebraska), and L* and b* values were moderately positively correlated (*R* = 0.5 in Michigan, *R* = 0.3 in Nebraska).

**TABLE 1:** Descriptive statistics of L*a*b* values of the Yellow Bean Collection

**Trait**	**Environment–year**	**Min**.	**Max**.	**Median**	**Mean**	**SD**
L*	MI 2018	27.6	84.6	68.8	66.7	10.2
	MI 2019	31.0	84.5	70.7	67.8	9.7
	NE 2018	33.5	85.3	71.6	69.9	10.0
	NE 2019	31.9	86.0	73.1	71.7	9.8
a*	MI 2018	−4.7	30.6	8.0	9.4	8.0
	MI 2019	−3.5	32.1	8.4	9.9	8.0
	NE 2018	−5.9	33.2	7.2	8.2	9.5
	NE 2019	−5.6	35.0	5.8	7.4	9.7
b*	MI 2018	11.9	62.1	47.2	45.5	9.1
	MI 2019	12.6	62.2	47.8	46.9	9.0
	NE 2018	11.9	60.3	43.5	44.5	9.6
	NE 2019	12.3	63.6	45.5	45.7	10.1

A total of 206 genotypes, excluding dark‐colored seeds, were evaluated for susceptibility to postharvest darkening by the UV test. The UV test distinguished whether a sample darkened or not but did not distinguish nondarkening from slow‐darkening samples. As a result, 71 were non‐ or slow darkening, and 135 genotypes showed a darkening phenotype (Table 2). The majority of Amarillo light and canary genotypes were darkening. All of Manteca were non‐ or slow darkening. ‘Prim’ Manteca bean was reported to contain no tannins (Beninger et al., 1998) and showed a *jj* genotype at the *J* locus, which would result in nondarkening (Bassett, 1999); therefore, Mantecas are expected to be nondarkening. For Mayocoba, 39 out of the 54 genotypes were slow‐ or nondarkening, and 14 of these were U.S. breeding lines. Because consumers highly value non‐ and slow‐darkening seeds, as this trait is associated with freshness and shorter cooking time (Erfatpour et al., 2018; de Cássia Silva et al., 2018; Wiesinger et al., 2021), information on the postharvest darkening of the YBC is useful in developing and selecting yellow beans that meet consumers’ expectations.

**TABLE 2:** Postharvest darkening of the Yellow Bean Collection genotypes by seed type

**Seed type**	**Non‐ or slow darkening**	**Darkening**	**Total**
Amarillo (dark)	0	2	2
Amarillo (light)	4	38	42
Beige	0	6	6
Brown	0	3	3
Canary	10	64	74
Green‐yellow	3	3	6
Manteca	11	0	11
Mayocoba	39	15	54
White	3	0	3
Amarillo dark and light mottled	0	1	1
Beige with brown stripes	0	3	3
White and red mottled	1	0	1
Total	71	135	206

A total of 258 YBC genotypes grown in MI2019 were classified based on their hilum ring and corona colors. Tables 3 and 4 show the number of genotypes in each category of hilum ring and corona colors by seed type. All the Manteca, Mayocoba, and white beans had a yellow hilum ring, and Amarillos, beige, brown, Canary, and green–yellow types had a dark hilum ring. For corona colors, Canary and Manteca types had dark corona, and Mayocoba type had light or yellow corona (Table 4), consistent with their characteristic seed appearance. Some Amarillos had light or yellow corona, and others had dark corona. Fifty‐three out of the 71 non‐ or slow‐darkening genotypes had a yellow hilum ring, and the other 18 had a dark hilum ring (Supplemental Table S2). The *J* gene is involved in both postharvest darkening and dark hilum ring color, and individuals with *jj* genotype are nondarkening (Elsadr et al., 2011; Junk‐Knievel et al., 2008). The *Z* gene is also involved in hilum ring color expression, and *Z* produces a dark hilum ring regardless of the *J* genotype (Bassett et al., 1999). Therefore, the 18 slow‐ or nondarkening genotypes that had a dark hilum ring must have *J* or *Z* unless other unreported genes are involved in hilum ring color expression. As expected, the 15 darkening genotypes that had yellow hilum rings were Mayocobas. Mayocobas carry the *gy* gene, which expresses yellow hilum ring regardless of the *J* genotype (Bassett, 2002). Forty‐eight genotypes were non‐ or slow‐darkening genotypes and had light or yellow corona (Supplemental Table S3). All the 11 Mantecas were among the non‐ or slow‐darkening genotypes with dark corona, and all the six beige genotypes were among the darkening genotypes with light or yellow corona. Genotypes of other seed types had different corona color and postharvest darkening (Supplemental Table S4). Mayocobas with yellow corona must be carrying *gy*, which expresses greenish‐yellow corona color (Bassett, 2002). The 11 genotypes that had yellow hilum ring and the dark corona (Supplemental Table S4) were all Mantecas, showing the uniqueness of this market class. ‘Prim’ Manteca had *v^lae^*, which gives dark corona (Bassett, 1999); thus, the dark corona of the 11 Manteca genotypes likely is due to *v^lae^*.

**TABLE 3:** Hilum ring (HR) color of the Yellow Bean Collection genotypes by seed type

**Seed type**	**Yellow HR**	**Dark HR**	**Total**
Amarillo (dark)	0	36	36
Amarillo (light)	0	42	42
Beige	0	6	6
Brown	0	7	7
Canary	0	83	83
Green‐yellow	0	6	6
Manteca	11	0	11
Mayocoba	56	0	56
White	3	0	3
Amarillo dark and light mottled	0	1	1
Amarillo (dark) with brown mottles	0	1	1
Amarillo (dark) with brown stripes	0	2	2
Beige with brown stripes	0	3	3
White and red mottled	0	1	1
Total	70	188	258

**TABLE 4:** Corona color of the Yellow Bean Collection genotypes by seed type

**Seed type**	**Light or yellow corona**	**Dark corona**	**Total**
Amarillo (dark)	17	19	36
Amarillo (light)	28	14	42
Beige	6	0	6
Brown	1	6	7
Canary	0	83	83
Green‐yellow	3	3	6
Manteca	0	11	11
Mayocoba	56	0	56
White	3	0	3
Amarillo dark and light mottled	1	0	1
Amarillo (dark) with brown mottles	1	0	1
Amarillo (dark) with brown stripes	2	0	2
Beige with brown stripes	3	0	3
White and red mottled	1	0	1
Total	122	136	258

### 3.2 G × E effects

The effects of genotype, environment, year, and interaction effects for the L*a*b* values are shown in Table 5. The genotype effect was significant for all the color values and postharvest darkening. The environment, G × E, and G × E × year effects for L*, a*, and b* values were significant, indicating that there is a G × E interaction for the color values. The genotype × year interaction was significant for a*, suggesting that a* of the seed colors in the YBC varied between years. However, the variation of a* as a result of year seems minimal because the ranges of a* were almost the same across the years and environments (Table 2) and the a* of the genotypes grown in each year were highly correlated (*R* ≥ 0.97). The heritability estimates of the color values were high (*R* ≥ 0.93; Table 5). This finding is consistent with other studies that reported high heritability of L*a*b* values with populations that had yellow or carioca bean as parental lines (*H* ^2^ > 0.73; Arns et al., 2018; Bassett et al., 2021; Possobom et al., 2015; Ribeiro et al., 2019). High heritability estimates of the color values indicate that genetic variance predominates in total phenotypic variance; thus, gain from selection is expected to be high.

**TABLE 5:** The *p‐*values for the factor effects on L*a*b* values and postharvest darkening traits

**Term**	L*	a*	b*	**Darkening** ^a^
Genotype (G)	<.0001	<.0001	<.0001	<.0001
Environment (E)	<.0001	<.0001	.046	–
Year (Y)	<.0001	.086	<.0001	–
G × E	<.0001	<.0001	<.0001	–
G × Y	.42	.015	1	–
E × Y	.026	<.0001	.84	–
G × E × Y	<.0001	<.0001	<.0001	–
*H* ^2^ ^b^	.99	.98	.93	–

^a^ Postharvest darkening was measured in Michigan in 2019.

^b^ Broad‐sense heritability.

### 3.3 Principal component analysis

**FIGURE 3:**
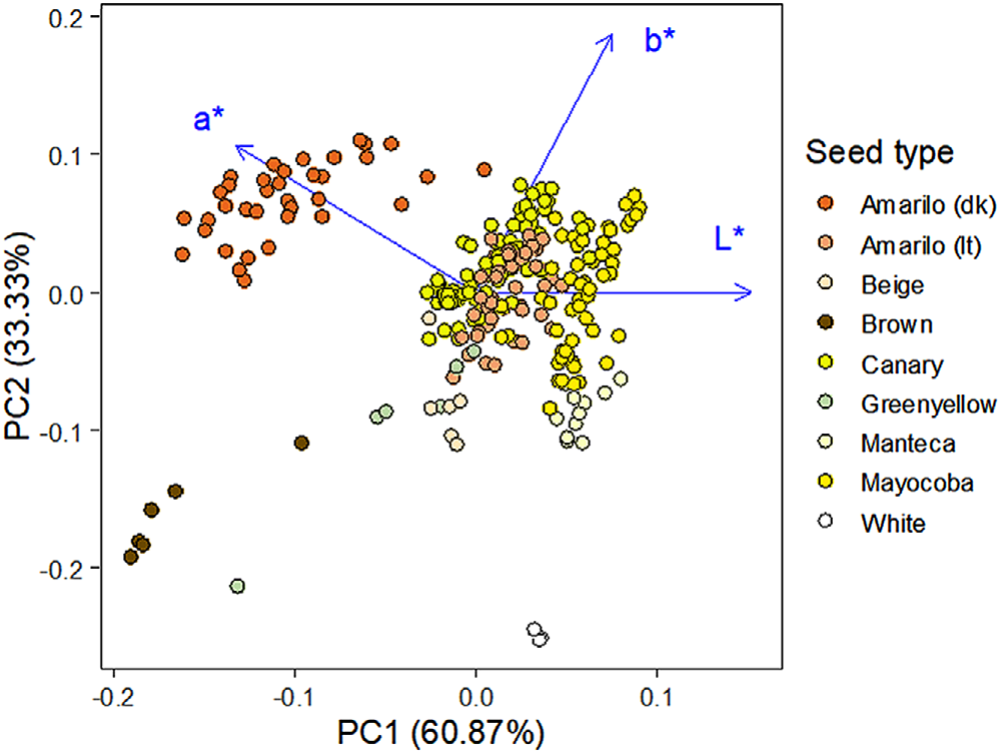
Biplot of principal component (PC) analysis of the L*a*b* values of the seed coat of the Yellow Bean Collection genotypes

Best linear unbiased estimation of color values adjusted for environment and year effects were used for PC analysis (Figure 3). Principal component 1 separated brown and Amarillo dark beans from the rest of the market classes explaining >60% of the total variance. Principal component 2 separated genotypes based on b* values, explaining 33% of the total variance. Together, PC1 and PC2 explained 94% of the total variance, indicating that the color values of the YBC could be compressed to two dimensions instead of three without losing much information. This seemed to be because of the high negative correlation between L* and a* values (Supplemental Figure S2). The L* and a* values are correlated mainly because of the Amarillo dark genotypes having higher a* and lower L* values than the other market classes of the YBC. Amarillo light, Canary, and green–yellow genotypes overlapped with one another in the biplot (Figure 3), showing the similarity of seed colors of these color types, which corresponds to our observation. Manteca and Mayocoba bean formed a relatively distinct cluster characterized by higher L* values than other seed types. Overall, the large and continuous variability of color values, even within market classes, indicated that the YBC is a source of various yellow colors that can meet specific consumer preferences.

### 3.4 Color differences between environments

**FIGURE 4:**
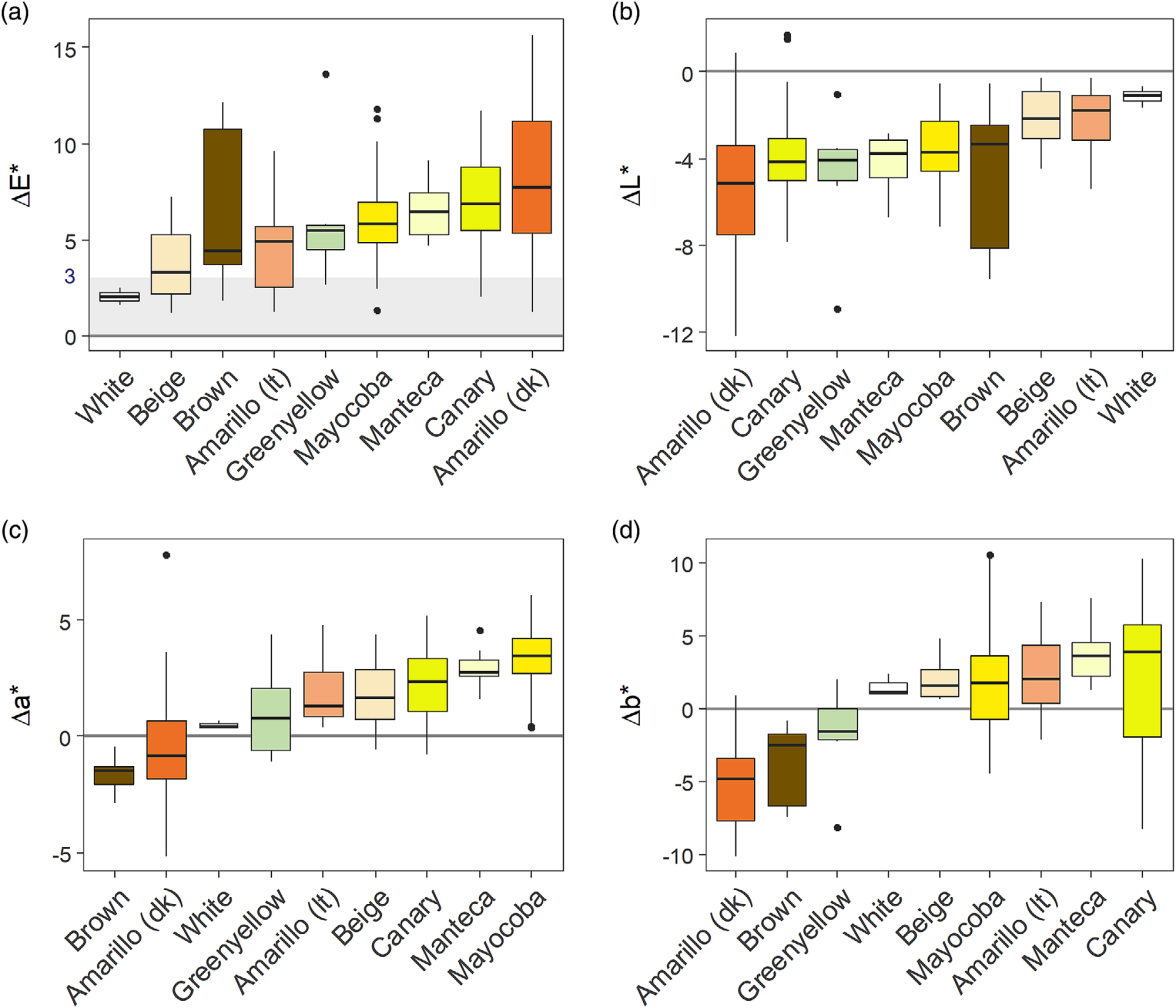
Color differences between MI and NE by seed type computed as differences between 2‐yr average of L*a*b* values in MI and NE. (a) ΔE*, (b) ΔL*, (c) Δa*, and (d) Δb*

Although the heritability for the L*a*b* values were high, G × E effect was significant (Table 5); therefore, the seed colors of Michigan‐ and Nebraska‐grown seed were compared via ΔL*, Δa*, and Δb*, such that positive values indicate higher color values of the Michigan‐grown seeds. The total differences were calculated as ΔE, a positive value that measures the degree of total color difference. The difference between two colors with ΔE of three or larger will be noticeable to the human eye (Haeghen et al., 2000). Many of the genotypes had ΔE larger than three (Figure 4a), indicating that their Michigan‐ and Nebraska‐grown seeds had noticeably different colors. The ΔL* value was negative for almost all the genotypes regardless of the seed types, indicating that Michigan‐grown seeds were darker than Nebraska‐grown seeds (Figure 4b). The absolute values of ΔL* values were larger for some of the dark‐colored seeds, such as brown and Amarillo dark types, so they were even darker in Michigan vs. Nebraska than the lighter‐colored yellow classes such as beige and Amarillo light. It agreed with previous literature and our observation that beans grown under humid conditions tend to produce darker seeds than those grown under dry conditions (Osorno et al., 2018; Possobom et al., 2015). Higher seed coat lightness (L*) was also reported when a black × carioca population was grown in a dry season than in a rainy season (Possobom et al., 2015). Almost all the Amarillo light, Beige, Canary, Manteca, and Mayocoba beans had positive Δa* values, indicating that the seeds produced in Michigan were redder (Figure 4c). Amarillo dark and brown beans had lower Δb* values, meaning that they were less yellow in Michigan than in Nebraska (Figure 4d). Mayocoba and Canary had varying Δb* values. Light‐colored beans, such as beige and Manteca types, had higher b* values, thus yellower in Michigan. Some Mayocoba‐type beans tend to produce paler colors under cool and wet fall in the U.S. Midwest (Kelly et al., 2021). Indeed, 17 Mayocobas had negative Δb*, meaning that Michigan‐grown beans were less yellow than Nebraska‐grown beans. The other 33 Mayocobas, however, had positive Δb*, which means yellower seeds were produced in Michigan, highlighting the importance of evaluating the color expression of Mayocoba beans in their target environments. Various factors could cause the difference in seed colors, such as humidity, rain, shorter exposure to sunlight, and increased pressure of certain diseases that favor moist conditions. Postharvest darkening was associated with the decrease in polymerization of proanthocyanidins in a regular‐darkening pinto bean (Beninger et al., 2005) and is characterized by the presence of proanthocyanidins and their precursors in regular‐darkening cranberry bean (Chen et al., 2015), but further studies are needed to comprehensively understand the relationships between growing conditions, seed color, and polyphenolic profiles.

It was hypothesized that the genotypes that had darker seeds in Michigan (lower ΔL*, Δa*, or Δb* values) might be darkening types if darkening was already initiated before the images were taken. However, no clear separation in the color difference between darkening and non‐ or slow‐darkening YBC lines was observed except for the b* values of Canary (Supplemental Figure S3). Canary bean with higher yellowness in Michigan were darkening, whereas non‐ or slow‐darkening Canary bean had lower yellowness in Michigan. Flavonoids, kaempferols in specific, are present in the seed coat of Canary bean (Hart et al., 2020), and it is possible that kaempferols present in darkening Canary bean grown in Michigan had started forming kaempferol‐catechin adduct, which increased in regular‐darkening pinto bean after darkening (Beninger et al., 2005).

### 3.5 Genome‐wide association analysis

**FIGURE 5:**
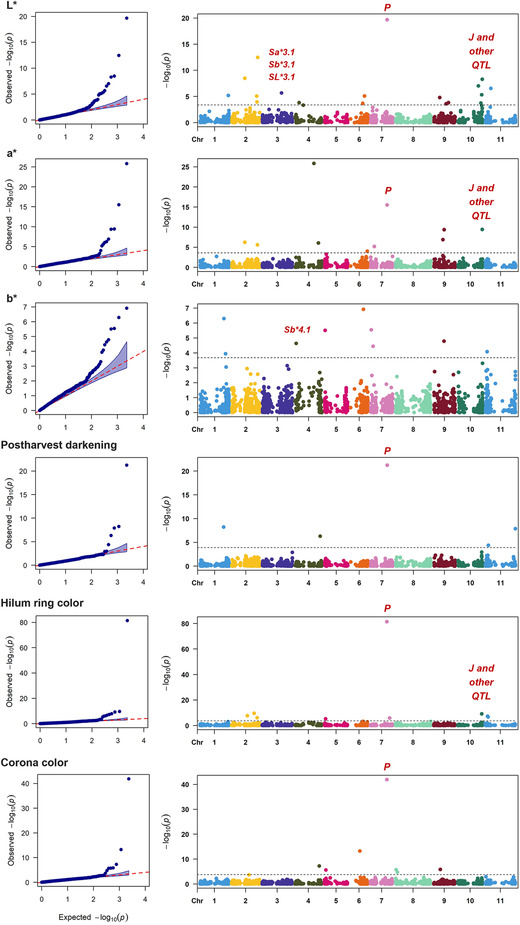
Quantile–quantile and Manhattan plots for L*, a*, b*, postharvest darkening, hilum ring color, and corona color. The gray dotted lines are false discovery rate‐adjusted threshold at α = 0.05. *P* is the ground factor gene for seed coat color, of which *p^sd^* allele confers slow darkening trait (Islam et al., 2020; McClean et al., 2018). Sb*.4.1, Sa*.3.1, Sb*.3.1, SL*.3.1, quantitative trait loci reported by Bassett et al. (2021). *J* and other QTL on Pv10 are the *J* gene and previously found QTL or significantly associated SNPs. *J* is the gene responsible for postharvest darkening (Elsadr et al., 2011). All the significant single nucleotide polymorphisms in this study for L* were within the ranges of Sa*.10.1, Sb*.10.1, SL*.10.1, and ND.10.1 (Bassett et al., 2021). Chr10pos42388055 was within the range of L*10.1BB (I) (Bornowski et al., 2020). Chr10pos42388055 was within the range of a*10.1BB (I) (Bornowski et al., 2020)

The Manhattan and quantile–quantile plots for the L*a*b* values, postharvest darkening, and hilum ring and corona colors are shown in Figure 5. Eighteen SNPs were significantly associated with L* value, 10 with a*, nine with b*, five for postharvest darkening, nine for hilum ring color, and seven for corona color (Supplemental Table S5). Three SNPs were significant for multiple traits: Chr02pos23133217 for a* and L*; Chr07pos29169848 for a*, L*, postharvest darkening, hilum ring color, and corona colors; and Chr10pos43341167 for a* and L*. Several SNPs were in proximity. Chr01pos41574533 for postharvest darkening and Chr01pos42211164 for b* were 637 kb apart. Chr02pos44319551 for L* and Chr02pos44931551 for L* were 612 kb apart. Chr02pos45646751 for hilum ring color, Chr02pos45663695 for a*, and Chr02pos46287738 for L* were all within the range of a 641‐kb region. Chr05pos2078923 for hilum ring color and Chr05pos2408324 for corona color were 329 kb apart. Chr09pos18409319 for b* and Chr09pos18733021 for a* were 324 kb apart. Chr10pos42388055 for L* and Chr10pos42797440 for hilum ring color were 409 kb apart, and the latter was 544 kb apart from Chr10pos43341167 for a* and L*. Chr11pos4985949 for postharvest darkening and Chr11pos5038396 for hilum ring color were 52 kb apart. The phenotypic effect was larger for the SNPs for L* than a* and b*, reflecting the wider distribution of L* (Table 1).

Chr01pos50086405 on Pv01 was in a region where disease resistance genes are clustered, specifically for *Colletotrichum lindemuthianum* (Kelly & Bornowski, 2018). Polyphenolics play an important part in plant defense mechanisms against pests and pathogens (Bennett & Wallsgrove, 1994; Islam et al., 2004). If a gene is involved in the expression of seed colors important for consumers and disease resistance important for growers, the breeders’ job would be to select genotypes that carry favorable alleles for both traits. It could be challenging and merit further research because an instance is reported that purple seed coat color and bean common mosaic virus resistance on Pv02 are tightly linked (or caused by a pleiotropic effect) and that it was not possible to introduce the resistance to other color types (Temple & Morales, 1986). Despite being beneficial in insect control, some polyphenols may act as an iron absorption inhibitor and adversely affect iron bioavailability (Hart et al., 2017); therefore, breeders need to balance the benefits and tradeoffs.

Some of the significant SNPs on Pv03, Pv04, and Pv10 in this study were in the QTL regions found in QTL studies for seed coat color: one with a pale lemon‐yellow × green‐yellow bean recombinant inbred line population (Bassett et al., 2021) and one with a black bean population (Bornowski et al., 2020), respectively (Figure 5). These SNPs will be a useful reference in future studies on yellow color expression in common bean.

Chr07pos29169848 was significant for L*, a*, postharvest darkening, hilum ring color, and corona color with low *p* values and large phenotypic effects (−10.0 for L*, +9.2 for a*, +0.59 for postharvest darkening, +0.90 for hilum ring color, and +0.64 for corona color; Supplemental Table S5). The minor allele group genotypes at this SNP had a larger L*, a lower a*, a higher percentage of genotypes resistant to darkening, and light‐colored hilum ring and corona (Supplemental Table S5). Intriguingly, all the minor allele group genotypes (47 genotypes for L*, a*, b*, hilum ring color, and corona color; 45 for postharvest darkening) were Mayocoba at this SNP. Among them, 22 were from the United States or Canada, and another 22 were from International Center for Tropical Agriculture, Colombia, whereas Mayocoba genotypes from other parts of the world such as Africa, Europe, and Mexico were found in the major allele group. Since the breeding materials from International Center for Tropical Agriculture and those from the United States are closely related, this SNP may indicate the importance of color for the selection of Mayocoba type. This SNP is located near the ground factor *P* gene (395 kb apart); the dominant *P* allele is necessary for seed coat color expression by other color genes (Bassett & Miklas, 2007; McClean, 2002; McClean et al., 2018). A QTL study with a Mayocoba × white bean population may reveal the potential role of the *P* gene (or another gene tightly linked to *P*) in giving the greenish‐yellow color in some of the Mayocoba beans. This SNP was also significant for postharvest darkening, and 36 out of the 45 Mayocoba genotypes in the minor allele group at this SNP were non‐ or slow‐darkening genotypes. Allele *p^sd^*, an allele of *P*, is involved in slow darkening in the presence of *J*; genotypes with homozygous recessive *p^sd^* shows a slower rate of darkening than regular‐darkening genotypes (Elsadr et al., 2011; Islam et al., 2020; Junk‐Knievel et al., 2008). The postharvest darkening genotype at Chr07pos29169848, background information, hilum ring color, corona color, and BLUE of the L*a*b* values of the YBC genotypes are summarized in Supplemental Table S6. Surprisingly, 10 Canaries were non‐ or slow‐darkening genotypes, although Canaries were reported to have proanthocyanidins (Hart et al., 2020). They had the major allele at this SNP same as Mantecas, which do not have proanthocyanidins and are considered to be carrying *j* (Beninger et al., 1998; Hart et al., 2020). The difference between the non‐ or slow‐darkening Canaries and Mantecas may be related to the other significant SNPs associated with postharvest darkening on Pv01, Pv04, and Pv11 (Supplemental Table S5).

The Chr08pos3300438 associated with corona color on Pv08 was 69 kb apart from the *Gy* gene. The *Gy* gene is considered to be responsible for the greenish‐yellow seed coat, hilum ring, and corona colors of Mayocoba type (Bassett, 2002). However, the minor allele group of this SNP included 11 Amarillos, two beige, and one white types that had light or yellow corona, and Mayocobas were all in the major allele group. The role of the *gy* gene in non‐Mayocoba types is yet to be researched. None of the significant SNPs were close to known color genes such as *Z*, *G*, *B*, and *V* that interactively impart yellow to brown seed coat, hilum ring, and corona colors (Bassett, 2007); however, despite the >2 Mbp distance, some SNPs associated with L*, a*, and corona color were on the same chromosomes as the color genes *B* on Pv02, *G* on Pv04, and *V* on Pv06. There is no preceding study that quantitatively measured yellow seed color besides Bassett et al. (2021), thus further investigation will be necessary on genes and their interactions that produce the wide diversity of yellow colors.

The region of a candidate *J* gene for brown hilum ring color and postharvest darkening has been narrowed down to 41,141,057–41,591,985 bp on Pv10 (Erfatpour et al., 2018), but Chr10pos42797440 associated with hilum ring color was ∼1.2 Mbp away from that region. A higher marker density and more SNPs closer to the *J* gene would help investigate the hilum ring color and postharvest darkening of yellow beans. Gene *J* was detected in this study for postharvest darkening but just below the significance threshold: Chr10pos42038959 on Pv10 near the *J* (447 bp apart) had a false discovery rate‐adjusted *p* value of .083. Nevertheless, a SNP (Chr10pos42797440) associated with hilum ring color within a range of a previously found QTL for postharvest darkening, ND.10.1 (3.98–43.85 Mbp; Bassett et al., 2021), lends support to the role of this region containing *J*. Another consideration for hilum ring color is that *J* and other genes are interacting—*J* is involved in the expression of dark hilum ring by *v^lae^* with the absence of Z*^chr^* (Bassett et al., 1999; Bassett, 2003), thus, hilum ring color may not be only determined by the genotype at *J*. Interestingly, Chr10pos40877199 on Pv10 associated with L* was close to the *J* gene (264 kb apart). The *J* gene is involved in mature seed coat color development and postharvest darkening by regulating the biosynthesis pathways of polyphenolic compounds (Bassett, 2007; Elsadr et al., 2011; Erfatpour et al., 2018).

## 4 CONCLUSIONS

In this study, the seed coat color of yellow beans was measured by masking noise in bean images such as hilum ring, corona, background, and light reflection using machine‐learning. As the trained model automatically segments seed coat from noise, it provides an efficient means to extract L*a*b* values from the seed coat. A large phenotypic diversity was observed for the L*a*b* values of the seed coat of the YBC genotypes grown in Michigan and Nebraska, USA. Despite the high heritability estimate for the color values, the G × E effects were significant. Most of the YBC lines grown in Michigan and Nebraska had a noticeable color difference, and those grown in Michigan with high humidity were darker. Genome‐wide association analysis discovered SNPs associated with the color values, postharvest darkening, and hilum ring and corona colors of the YBC genotypes. The L*, a*, postharvest darkening, hilum ring color, and corona color mapped near the *P* (*P^sd^*) gene. The phenotypic diversity and machine‐learning‐aided image analysis of the seed coat color and other traits provide a resource to objectively evaluate and understand the seed morphology of beans, which will lead to the development of new cultivars that better meet consumers’ needs.

## AUTHOR CONTRIBUTIONS

Rie Sadohara: Data curation; Formal analysis; Investigation; Methodology; Validation; Visualization; Writing‐original draft. Yunfei Long: Formal analysis; Methodology; Software; Writing‐review & editing. Paulo Izquierdo: Investigation; Methodology; Writing‐review & editing. Carlos A. Urrea: Funding acquisition; Investigation; Methodology; Writing‐review & editing. Daniel Morris: Methodology; Software; Supervision; Writing‐original draft. Karen A. Cichy: Conceptualization; Funding acquisition; Project administration; Resources; Supervision; Writing‐review & editing.

## CONFLICT OF INTEREST

There are no conflicts of interest to be disclosed.

## Supporting information


**Supplemental Figure S1** Examples of diversity in seed morphology of the YBC.
**Supplemental Figure S2** Pearson correlation between two‐year average of L*a*b* in each environment (MI and NE).
**Supplemental Figure S3** Color differences **A**: ΔE*, **B**: ΔL*, **C**: Δa*, and **D**: Δb* between MI and NE by seed type and postharvest darkening.
**Supplemental Table S1** The number of genotypes phenotyped for color values and postharvest darkening.
**Supplemental Table S2** Number of genotypes in the postharvest darkening and hilum ring color categories.
**Supplemental Table S3** Number of genotypes in the postharvest darkening and corona color categories.
**Supplemental Table S4** Number of genotypes in the hilum ring and corona color categories
**Supplemental Table S5** SNPs significantly associated with L*, a*, b*, postharvest darkening, hilum ring color, and corona color.

**Supplemental Table S6** YBC phenotype and information.xlsx. The postharvest darkening, genotype at Chr07pos29169848, background information, hilum ring color, corona color, and Best Linear Unbiased Estimation of L*a*b* values of the Yellow Bean Collection genotypes. [Supplementary file]

**Supplemental Table S7** BLUE Lab Genotype.xlsx. The genotypic data of the Yellow Bean Collection phenotyped for the L*a*b* values. [Supplementary file]

**Supplemental Table S8** Postharvest darkening Genotype.xlsx. The genotypic data of the Yellow Bean Collection phenotyped for postharvest darkening. [Supplementary file]

**Supplemental Table S9** HR and corona colors Genotype.xlsx. The genotypic data of the Yellow Bean Collection phenotyped for hilum ring and corona colors. [Supplementary file]
