# Supplementary material for: Seed coat color genetics and genotype × environment effects in yellow beans via machine‐learning and genome‐wide association
Source: Plant Genome. 2021 Nov 24;15(1):e20173. doi: 10.1002/tpg2.20173 (PMC12806898; doi:10.1002/tpg2.20173)
Supplement: Supplementary file 1 [file TPG2-15-e20173-s001.pdf]

Seed Coat Color Genetics and G×E in a Yellow Bean Collection via Image Analysis Paired with Machine-Learning and GWAS

Rie Sadohara[1], Yunfei Long[2], Paulo Izquierdo[1], Carlos A. Urrea[3], Daniel Morris[2], Karen Cichy[1,4]

Affiliations:

1. Department of Plant, Soil and Microbial Sciences, Michigan State University, 1066 Bogue St., East Lansing, MI 48824, USA
2. Department of Electrical and Computer Engineering, Michigan State University, 428 S Shaw Ln, East Lansing, MI 48824, USA
3. Panhandle Research & Extension Center, University of Nebraska, 4502 Ave I, Scottsbluff, NE 69361, USA
4. Sugarbeet and Bean Research Unit, USDA-ARS, 1066 Bogue St., East Lansing, MI 48824, USA

SUPPORTING MATERIALS

Supplementary figures

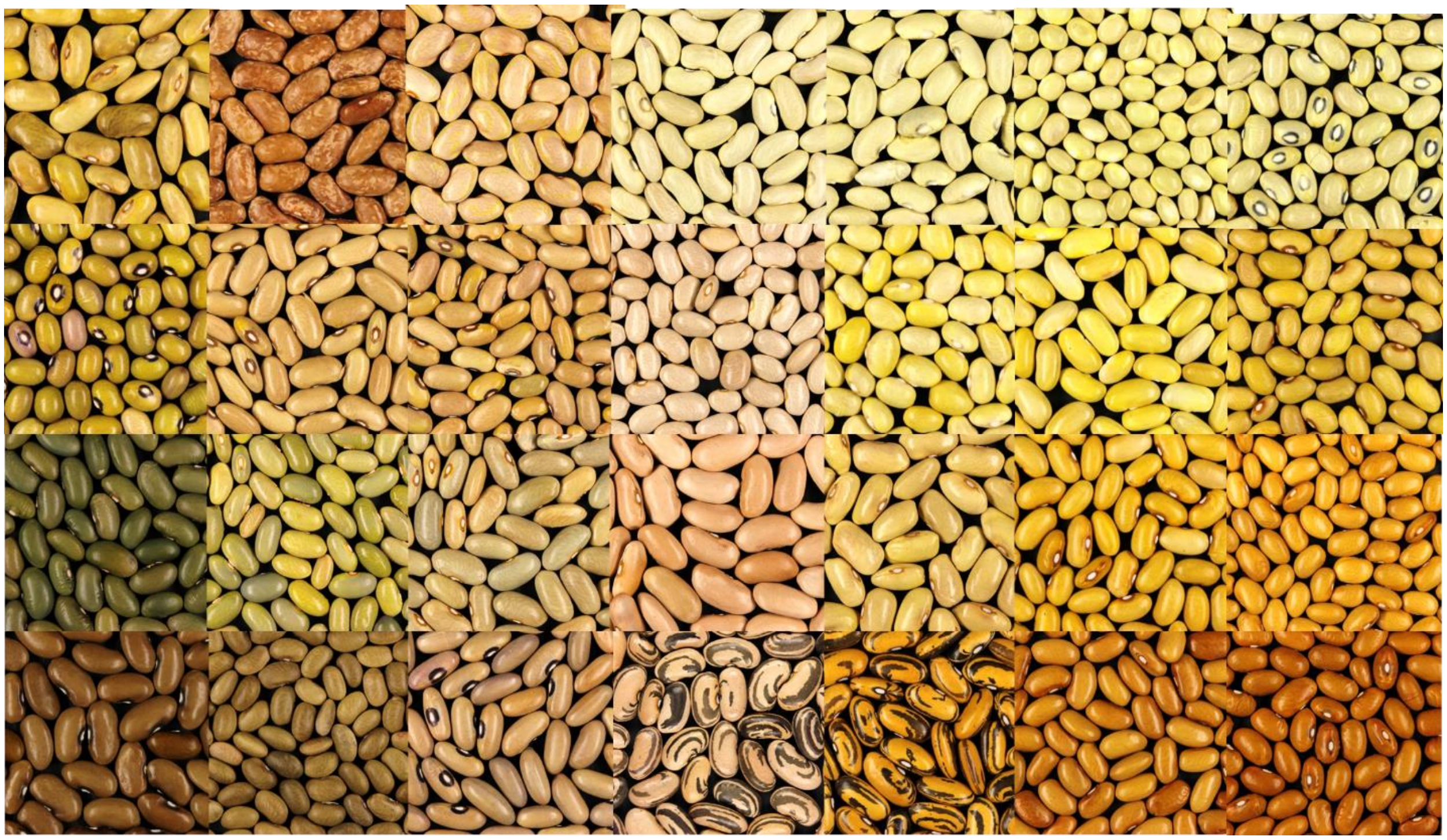

Figure S1. Examples of diversity in seed morphology of the YBC.

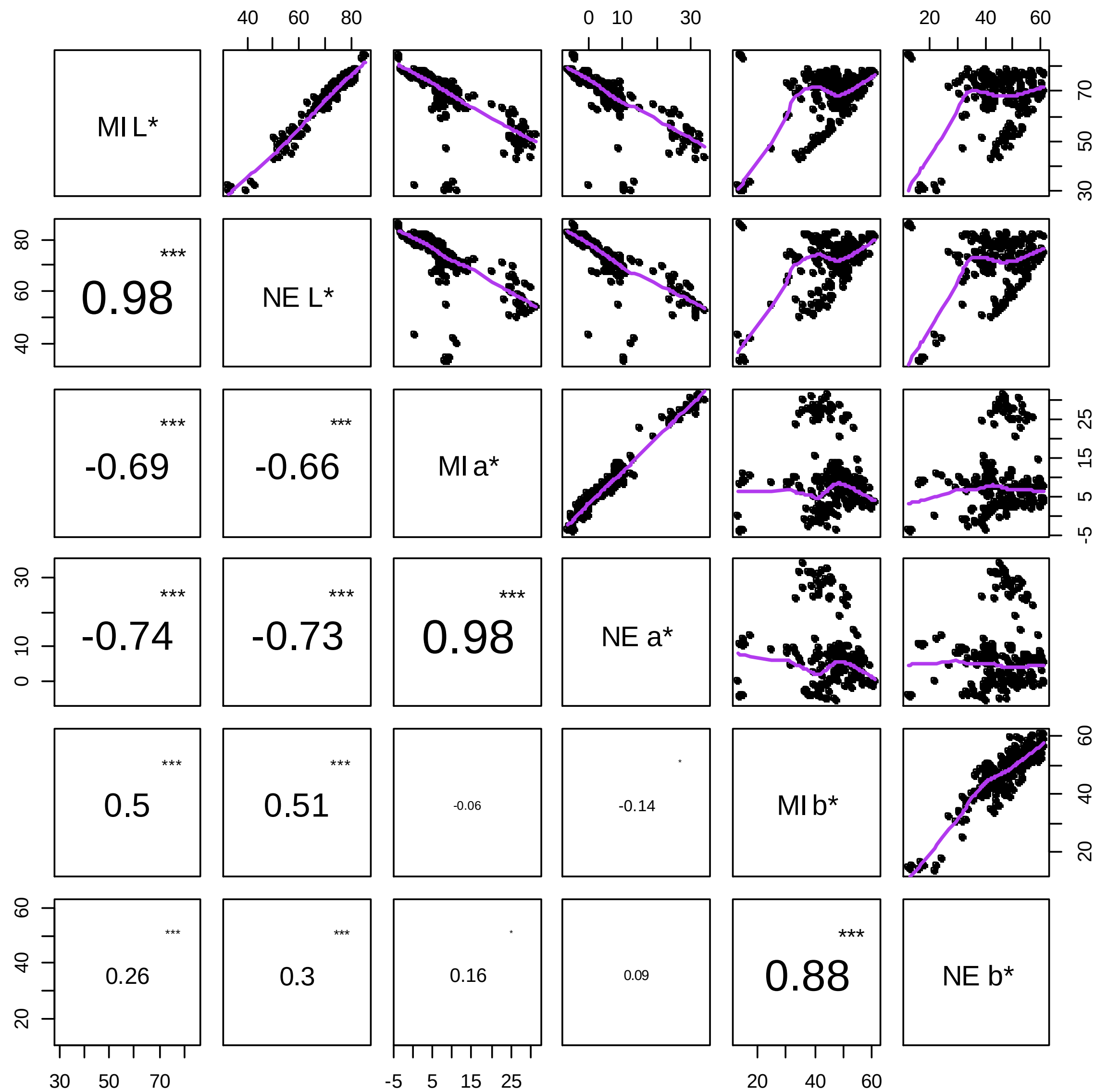

Figure S2. Pearson correlation between two-year average of L*a*b* in each environment (MI and NE).

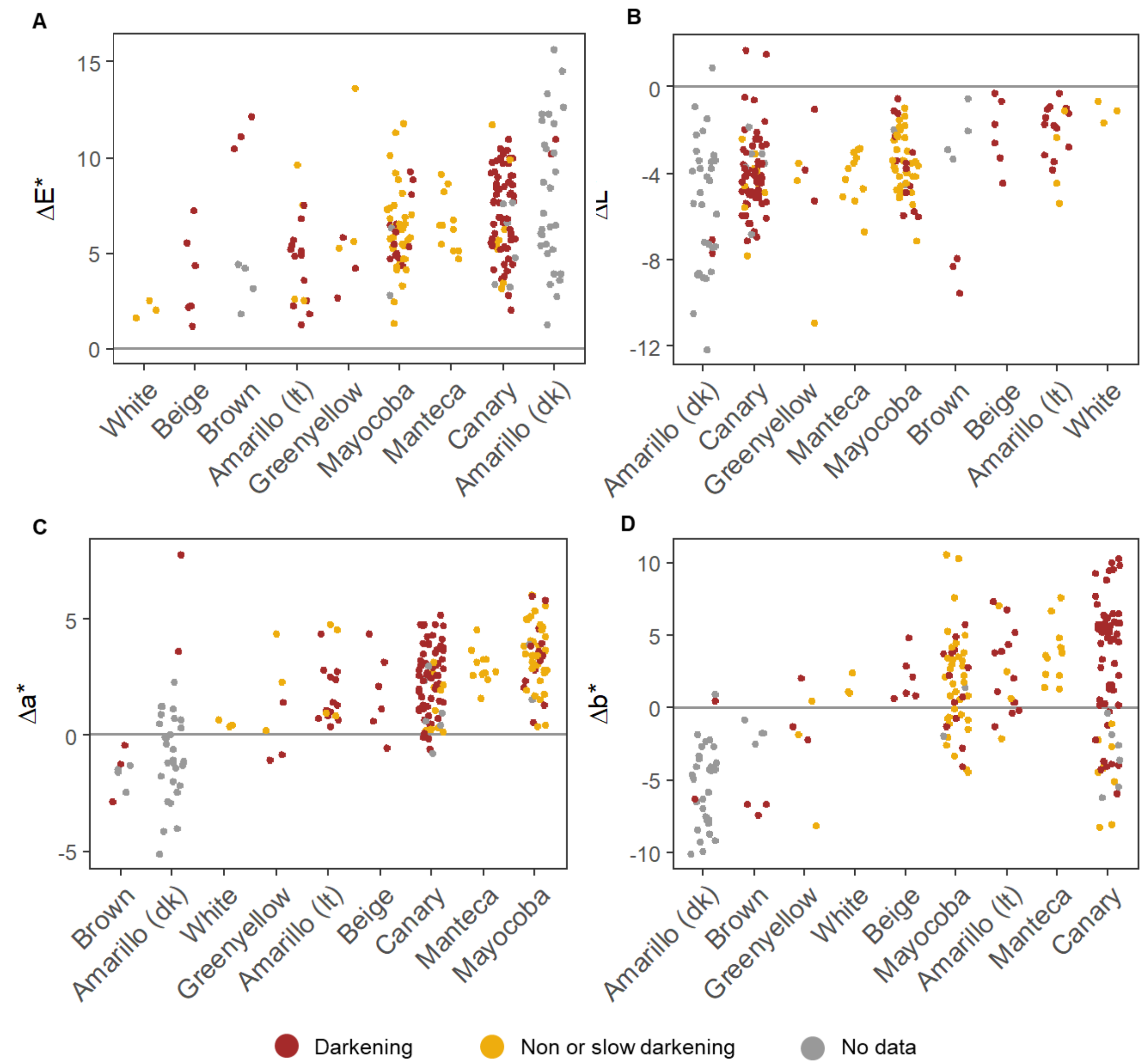

Figure S3. Color differences **A**: ΔE*, **B**: ΔL*, **C**: Δa*, and **D**: Δb* between MI and NE by seed type and postharvest darkening.

Supplementary tables

Table S1. The number of genotypes phenotyped for color values and postharvest darkening.

	Environment × Year	Total
L*, a*, b*	MI 2018	252
	MI 2019	250
	NE 2018	209
	NE 2019	195
Postharvest darkening	MI 2019	206
Hilum ring and corona colors	MI 2019	258

Table S2. Number of genotypes in the postharvest darkening and hilum ring color categories**.**

		Hilum ring		
		Yellow	Dark	Total
Postharvest darkening	Non or slow - darkening	53	18	71
	Darkening	15	120	135
	Total	68	138	206

Table S3. Number of genotypes in the postharvest darkening and corona color categories.

		Corona		
		Light/yellow	Dark	Total
Postharvest darkening	Non or slow - darkening	48	23	71
	Darkening	53	82	135
	Total	101	105	206

Table S4. Number of genotypes in the hilum ring and corona color categories.

		Corona		
		Light/yellow	Dark	Total
Hilum ring	Yellow	59	11	70
	Dark	63	125	188
	Total	122	136	258

Table S5. SNPs significantly associated with L*, a*, b*, postharvest darkening, hilum ring color, and corona color.

				------ Major allele ------			------ Minor allele ------				
SNP	Chr.	Position	FDR-adjusted *p*-values	Allele	n	Mean phenotypic value	Allele	n	Mean phenotypic value	MAF	SNP effect[a]
**L***											
Chr01pos50086405	1	50,086,405	1.6E-03	AA	230	70.0	TT	14	60.5	0.057	9.5
Chr02pos23133217	2	23,133,217	2.5E-06	AA	173	68.7	GG	59	71.1	0.254	-2.3
Chr02pos44319551	2	44,319,551	1.8E-03	CC	164	69.8	TT	58	68.7	0.261	1.0
Chr02pos44931551	2	44,931,551	1.9E-02	GG	164	68.6	TT	62	70.4	0.274	-1.8
Chr02pos46287738	2	46,287,738	3.9E-10	GG	240	70.1	TT	13	55.2	0.051	14.9
Chr03pos34038425	3	34,038,425	6.9E-04	TT	207	69.0	CC	11	70.9	0.050	-1.9
Chr04pos7036250	4	7,036,250	2.3E-02	AA	173	68.8	TT	67	71.4	0.279	-2.6
Chr06pos21948635	6	21,948,635	2.8E-02	TT	187	68.9	CC	31	73.3	0.142	-4.4
Chr06pos24955965	6	24,955,965	1.8E-03	CC	126	67.6	TT	81	72.7	0.391	-5.1
Chr07pos29169848	7	29,169,848	4.8E-17	TT	200	67.3	GG	47	77.3	0.190	-10.0
Chr09pos10759327	9	10,759,327	2.9E-03	GG	231	68.9	AA	13	75.2	0.053	-6.3
Chr09pos22109781	9	22,109,781	3.1E-02	GG	184	68.8	AA	42	72.4	0.186	-3.7
Chr09pos26420953	9	26,420,953	2.3E-02	TT	144	69.5	GG	59	70.4	0.291	-0.9
Chr10pos36447913	10	36,447,913	4.3E-05	AA	179	69.8	GG	37	69.6	0.171	0.2
Chr10pos40877199	10	40,877,199	2.5E-02	AA	144	68.4	GG	70	71.0	0.327	-2.6
Chr10pos42388055	10	42,388,055	1.3E-03	CC	195	70.4	AA	19	66.1	0.089	4.3
Chr10pos43341167	10	43,341,167	2.9E-06	CC	167	70.6	TT	54	67.7	0.244	2.9
Chr11pos9547435	11	9,547,435	1.1E-04	CC	220	70.4	TT	15	57.5	0.064	13.0
**a***											
Chr02pos23133217	2	23,133,217	2.2E-04	AA	173	8.5	GG	59	9.3	0.254	-0.8
Chr02pos45663695	2	45,663,695	6.8E-04	AA	133	8.8	TT	78	7.6	0.370	1.1
Chr04pos33747479	4	33,747,479	3.3E-23	GG	153	5.2	AA	90	14.2	0.370	-9.1
Chr04pos41620411	4	41,620,411	2.9E-04	CC	155	8.2	TT	57	8.9	0.269	-0.7
Chr06pos30013548	6	30,013,548	2.2E-02	GG	194	8.5	AA	53	8.9	0.215	-0.4
Chr07pos6344071	7	6,344,071	1.6E-03	AA	157	8.5	GG	50	8.4	0.242	0.1
Chr07pos29169848	7	29,169,848	3.4E-13	TT	200	10.5	GG	47	1.3	0.190	9.2
Chr09pos16476454	9	16,476,454	6.0E-05	TT	166	7.5	CC	51	10.3	0.235	-2.8
Chr09pos18733021	9	18,733,021	2.5E-07	TT	158	9.6	AA	54	7.8	0.255	1.8
Chr10pos43341167	10	43,341,167	2.5E-07	CC	167	6.8	TT	54	13.4	0.244	-6.6
**b***											
Chr01pos42211164	1	42,211,164	5.8E-04	GG	167	45.0	AA	44	47.7	0.209	-2.7
Chr01pos45147107	1	45,147,107	2.9E-02	CC	190	46.9	TT	17	40.0	0.082	6.9
Chr04pos1909564	4	1,909,564	8.8E-03	TT	201	47.3	AA	28	40.5	0.122	6.8
Chr05pos817906	5	817,906	1.8E-03	GG	123	48.4	AA	97	43.4	0.441	5.0
Chr06pos23037583	6	23,037,583	2.8E-04	GG	203	44.9	AA	32	51.5	0.136	-6.6
Chr07pos962487	7	962,487	1.8E-03	CC	200	45.5	AA	21	49.1	0.095	-3.6
Chr07pos4181998	7	4,181,998	1.2E-02	TT	187	46.2	CC	33	43.7	0.150	2.6

Chr09pos18409319	9	18,409,319	7.5E-03	CC	214	45.9	TT	13	45.7	0.057	0.2
Chr11pos2684350	11	2,684,350	2.4E-02	GG	116	47.5	AA	114	44.8	0.496	2.7
**Postharvest darkening**											
Chr01pos41574533	1	41,574,533	6.7E-06	TT	192	0.68	AA	14	0.29	0.068	0.40
Chr04pos44665073	4	44,665,073	2.8E-04	CC	165	0.68	GG	21	0.48	0.113	0.21
Chr07pos29169848	7	29,169,848	1.3E-18	TT	155	0.79	GG	45	0.20	0.225	0.59
Chr11pos4985949	11	4,985,949	2.0E-02	GG	181	0.67	TT	16	0.44	0.081	0.24
Chr11pos53315933	11	53,315,933	9.9E-06	TT	133	0.56	CC	37	0.89	0.218	-0.34
**Hilum ring color**											
Chr02pos28089361	2	28,089,361	1.0E-05	TT	199	0.73	CC	28	0.71	0.123	0.01
Chr02pos40280426	2	40,280,426	2.8E-07	TT	172	0.70	CC	62	0.77	0.265	-0.08
Chr02pos45646751	2	45,646,751	2.6E-04	TT	169	0.67	AA	73	0.86	0.302	-0.19
Chr05pos2078923	5	2,078,923	1.4E-03	AA	164	0.67	GG	48	0.90	0.226	-0.23
Chr07pos29169848	7	29,169,848	9.1E-79	TT	205	0.90	GG	47	0.00	0.187	0.90
Chr07pos34201349	7	34,201,349	3.9E-04	CC	204	0.69	TT	11	1.00	0.051	-0.31
Chr10pos42797440	10	42,797,440	5.5E-07	AA	146	0.70	GG	75	0.79	0.339	-0.09
Chr11pos4112749	11	4,112,749	2.4E-05	TT	204	0.75	GG	16	0.38	0.073	0.38
Chr11pos5038396	11	5,038,396	7.6E-05	AA	226	0.73	GG	21	0.62	0.085	0.12
**Corona color**											
Chr04pos43635399	4	43,635,399	4.5E-05	GG	210	0.50	CC	14	0.79	0.063	-0.29
Chr05pos2408324	5	2,408,324	8.8E-04	AA	168	0.68	TT	40	0.23	0.192	0.45
Chr06pos17242131	6	17,242,131	6.9E-11	TT	200	0.60	AA	20	0.30	0.091	0.30
Chr07pos29169848	7	29,169,848	2.9E-39	TT	205	0.64	GG	47	0.00	0.187	0.64
Chr08pos1159204	8	1,159,204	8.6E-04	AA	196	0.54	GG	15	0.33	0.071	0.21
Chr08pos3300438	8	3,300,438	6.4E-03	GG	222	0.61	AA	14	0.00	0.059	0.61
Chr09pos12073563	9	12,073,563	8.6E-04	TT	190	0.64	CC	25	0.16	0.116	0.48

[a]SNP effect is the difference between the mean cooking times of the YBC lines with the major and minor alleles.

Table S6 YBC phenotype and information.xlsx. The postharvest darkening, genotype at Chr07pos29169848, background information, hilum ring color, corona color, and Best Linear Unbiased Estimation of L*a*b* values of the Yellow Bean Collection genotypes.

Table S7 BLUE Lab Genotype.xlsx. The genotypic data of the Yellow Bean Collection phenotyped for the L*a*b* values.

Table S8 Postharvest darkening Genotype.xlsx. The genotypic data of the Yellow Bean Collection phenotyped for postharvest darkening.

Table S9 HR and corona colors Genotype.xlsx. The genotypic data of the Yellow Bean Collection phenotyped for hilum ring and corona colors.
